# First report and genetic characterization of bovine torovirus in diarrhoeic calves in China

**DOI:** 10.1186/s12917-020-02494-1

**Published:** 2020-08-05

**Authors:** Zhihai Shi, Wenjia Wang, Chaoxi Chen, Xiaozhan Zhang, Jing Wang, Zhaoxue Xu, Yali Lan

**Affiliations:** 1 Institute of Animal Husbandry and Veterinary Medicine, Henan Academy of Agricultural Sciences, Zhengzhou, 450002 Henan China; 2 Henan Key Laboratory of Farm Animal Breeding and Nutritional Regulation, Zhengzhou, 450002 Henan China; 3 College of Veterinary Medicine and Pharmaceutical Engineering, Henan University of Animal Husbandry and Economy, Zhengzhou, 450046 Henan China; 4 College of Life Science and Technology, Southwest University for Nationalities, Chengdu, 610041 Sichuan China

**Keywords:** Bovine torovirus, China, Calf diarrhoea, Beef, Dairy, Phylogenetic analysis

## Abstract

**Background:**

Coronaviruses are notorious pathogens that cause diarrheic and respiratory diseases in humans and animals. Although the epidemiology and pathogenicity of coronaviruses have gained substantial attention, little is known about bovine coronavirus in cattle, which possesses a close relationship with human coronavirus. Bovine torovirus (BToV) is a newly identified relevant pathogen associated with cattle diarrhoea and respiratory diseases, and its epidemiology in the Chinese cattle industry remains unknown.

**Results:**

In this study, a total of 461 diarrhoeic faecal samples were collected from 38 different farms in three intensive cattle farming regions and analysed. Our results demonstrated that BToV is present in China, with a low prevalence rate of 1.74% (8/461). The full-length spike genes were further cloned from eight clinical samples (five farms in Henan Province). Phylogenetic analysis showed that two different subclades of BToV strains are circulating in China. Meanwhile, the three BToV strains identified from dairy calves, 18,307, 2YY and 5YY, all contained the amino acid variants R614Q, I801T, N841S and Q885E.

**Conclusions:**

This is the first report to confirm the presence of BToV in beef and dairy calves in China with diarrhea, which extend our understanding of the epidemiology of BToVs worldwide.

## Background

Calf scours, also known as calf diarrhoea, is an enteric disease complex in young calves and is regarded as the leading cause of mortality in dairy calves, causing severe economic losses to the cattle industry worldwide. The causes of calf scours are varied and complex, including infectious and non-infectious factors. In recent decades, a large number of infectious agents have been identified to be associated with calf scours, including bacteria, viruses and parasites. Thus far, the viral pathogens that cause calf diarrhoea include bovine coronavirus (BCoV) [1], bovine rotavirus (BRV) [2], bovine viral diarrhoea virus (BVDV) [3], bovine torovirus (BToV) [4], bovine kobuvirus (BKoV) [5], bovine astrovirus (BAstV) [6], bovine norovirus (BNoV) [7], bovine enterovirus (BEV) and bovine nebovirus (BNeV) [8].

Torovirus (ToV) is a newly identified enveloped, single-stranded, positive-sense RNA virus that belongs to the subfamily *Toroviridae*in the family *Coronaviridae*, order *Nidovirales* [9]. The genome of ToV is 25–30 kb in length and contains two large open reading frames (ORFs) that encode two nonstructural proteins, ORF1a and ORF1ab, and four structural proteins, the spike (S), membrane (M), haemagglutinin-esterase (HE), and nucleocapsid (N) proteins [10].

Coronaviruses have drawn significant attention as a result of their pathogenicity toward humans and animals, as they usually cause significant respiratory and gastrointestinal diseases, such as severe acute respiratory syndrome (SARS), Middle East respiratory syndrome (MERS), porcine epidemic diarrhoea (PED), and transmissible gastroenteritis (TGE). Similar to other members of *Coronaviridae,* ToV can infect a wide range of animals, including humans. In recent decades, ToV has been identified in humans, horses, cattle, and pigs worldwide, and the clinical sign of ToV infection is gastroenteritis; however, its role remains unclear. BToV was first identified in America from diarrhoeic calves in 1979 [11]. Since then, BToV has been reported in France, South Africa, Costa Rica, Canada, Venezuela, Hungary, Austria, Japan, South Korea, Brazil and, in recent years, Turkey and Croatia, which suggests that it has become a worldwide problem [4, 12–27]. It is associated with respiratory and gastrointestinal diseases. An artificial inoculation trial also revealed the pathogenesis of BToV in cattle [11, 28]. Although these data strongly suggest that BToV is relevant to diarrhoea in cattle, to our knowledge, there have been no reports of BToV in China to date. Herein, our study investigated the presence and molecular characteristics of BToV in faecal samples from diarrhoeic calves in China. This report is the first to describe the presence of BToV in Chinese beef and dairy, and to analyse the genetic characteristics and phylogenetic relationships between the Chinese BToV, BToV reference strains and other representative toroviruses.

## Results

### BToV detection and coinfections

**Fig. 1:**
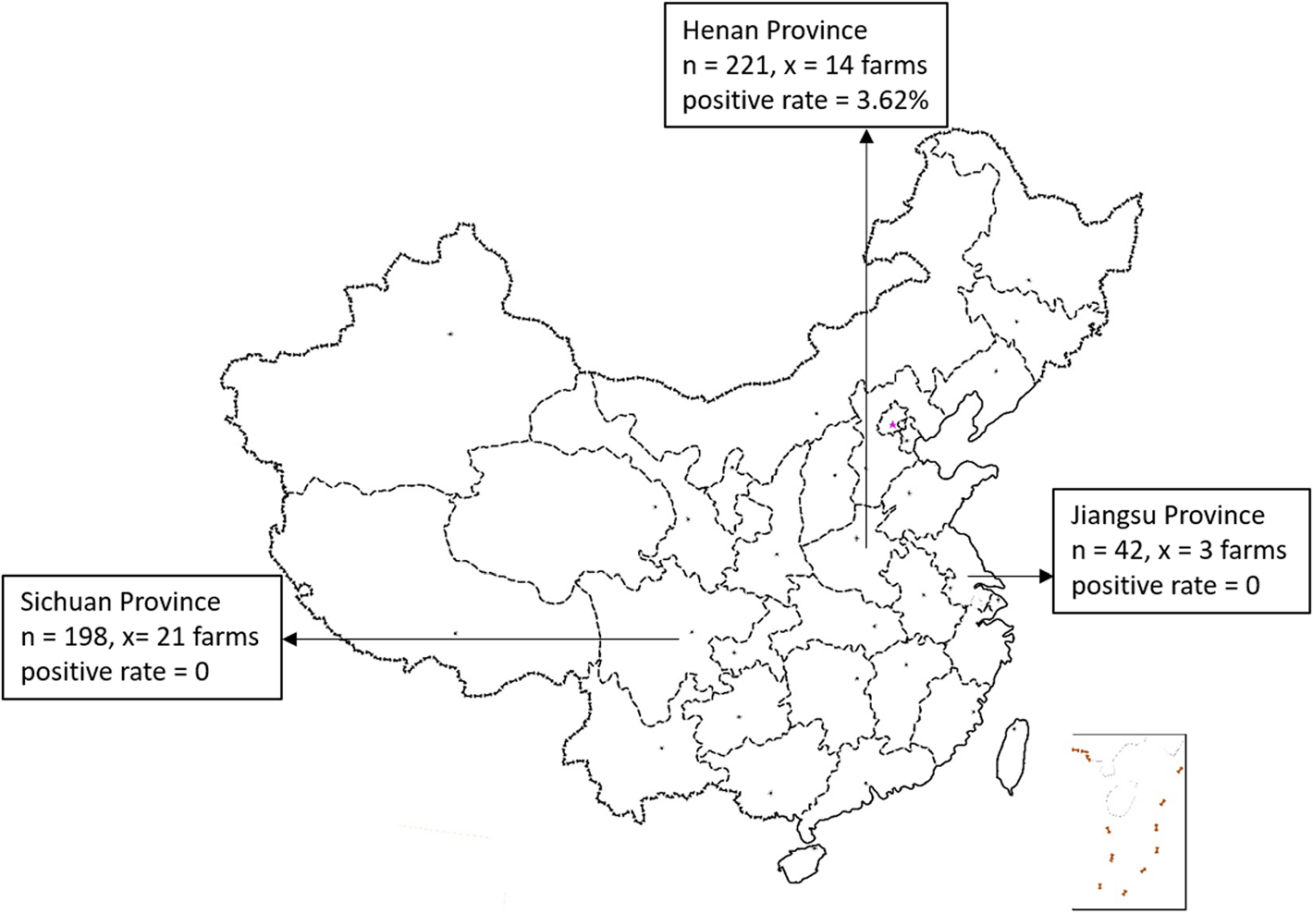
Number of calves and farms from three provinces in China, 2017–2019. The n values indicate the total number of samples in each province, the x values indicate the total number of farms in each province, and the positive rate indicates the BToVs positive rate. The map was created by MAPGIS (version 6.7)

A total of 461 faecal samples from calves with clinical signs of diarrhoea were detected by RT-PCR, and the results demonstrated that 1.74% (8/461) of the diarrhoeal samples were positive for BToV. Moreover, 5 out of the 38 farms were positive for BToV. Notably, BToV was found in diarrhoeal faecal samples from only Henan Province, and the overall detection rate was 3.62% (8/221) [4.07% (5/123) and 3.06% (2/98) in dairy calves and beef cavles, respectively] (Table 1, Fig. 1). The positive rate of farms was 35.7% (5/14). Detailed test results of the eight positive samples are shown in Table 2.

**Table 1:** The result of BToV detection in diarrhoea faecal samples

Area	Breed	Number of farms	Number of samples	Positive rate %
Henan		14	221	3.62 (8/221)
	dairy	7	123	4.07 (5/123)
	beef	7	98	3.06 (3/98)
Jiangsu		3	42	0
	dairy	2	24	0
	beef	1	18	0
Sichuan		21	198	0
	yak	21	198	0
Total		38	461	1.74 (8/461)

**Table 2:** The information of samples and test results

Name	Accession number	Enteric pathogens										
		BToV	BCoV	BRVA	BRVB	BRVC	BVDV	BKV	BAstV	BNebV	*Coccidium* species	*Cryptosporidium* species
2YY	MT364036	+	–	–	–	+	–	–	–	–	–	–
5YY	MT364037	+	+	–	–	–	–	–	–	–	–	–
3BY^a^	MT364038	+	–	–	–	–	–	–	–	–	–	–
6BY^a^	MT364039	+	–	–	–	–	–	–	–	–	–	–
2LSK	MT364040	+	+	–	–	–	–	–	–	–	–	–
18307^a^	MT364041	+	–	–	–	–	–	–	–	–	–	–
N4	MT364042	+	–	+	–	–	+	+	+	–	–	–
N7	MT364043	+	–	+	–	+	–	+	+	+	–	–

^a^Only detected BToV in our study

+ indicated positive for the organism, − indicated negative for the organism

### PCR amplification of the S genes

The full-length S genes were successfully cloned from 8 positive samples from 5 different farms in Henan Province. The newly determined sequences were deposited in GenBank under accession numbers MT364036 to MT364043 (Table 2).

### Molecular characterization of the S genes

The 8 S genes were 4755 bp in length, and each encoded a protein of 1585 amino acid (aa). Sequence comparisons showed that all 8 S genes shared 95.4–99.9% nt identity and 95.6–99.8% aa identity with each other. They also shared 94.3–97.7% nt identity and 95.1–98.4% aa identity with all 11 full-length BToV S genes available in GenBank and shared 92.0–92.9% nt identity and 93.6–95.3% aa identity with goat torovirus S genes (KR527150); however, they shared 73.7–75.9% nt identity and 75.8–79.6% aa identity with the human, equine and swine torovirus S genes (JQ860350, MG996765 and KM403390).

**Fig. 2:**
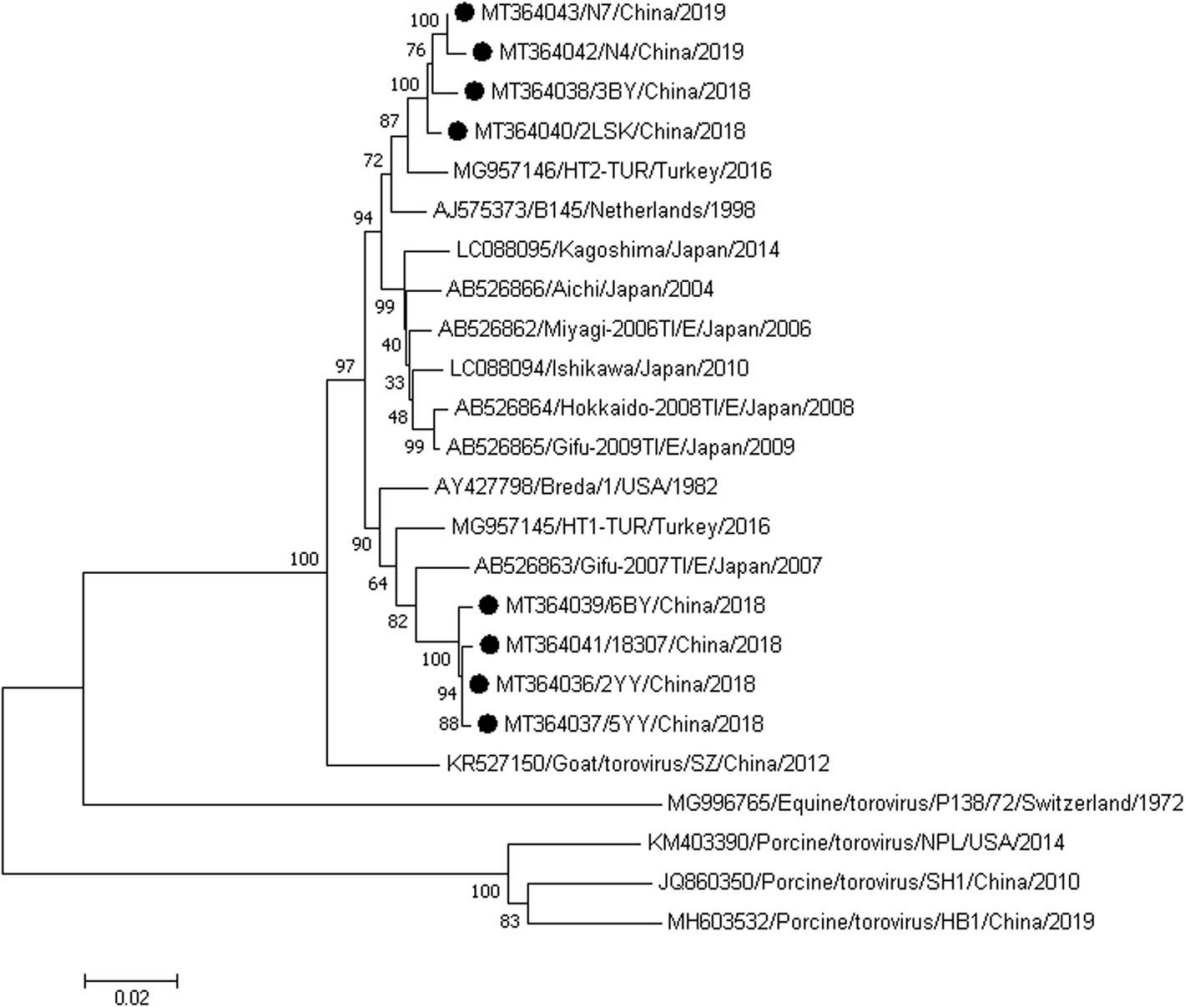
Phylogenetic analysis of BToV strains based on the amino acid sequences of their complete S genes. The phylogenetic tree was constructed by the neighbour-joining method with 1000 bootstrap replicates using MEGA 7.0 software. The black circles represent the new Chinese strains

Phylogenetic analyses were further performed for the newly identified Chinese BToV strains. The results showed that the 8 newly identified Chinese BToV strains were clustered in the same group with other BToVs and were distantly related to the ToV strains derived from human, equine and porcine sources. A phylogenetic tree based on the aa sequences of the complete S gene sequences using the neighbour-joining method demonstrated that the 8 S genes identified in this study were clustered into two different subclades of BToV strains, which clustered closely with the Turkish strain (MG957146) and the Japanese strain (AB526863) (Fig. 2).

Recombination events among the S genes of the BToV strains were further investigated using RDP4 software, but no correlations were detected. This result indicated that no recombination had occurred between the BToVs (data not shown). However, the available BToV S gene sequences are limited; therefore, there may not be enough data available to confirm this result. Therefore, further studies of BToVs are required.

Compared with the other BToV S gene sequences, 3/8 sequences identified in this study, all of which were obtained from dairy strains, had four identical aa variants (R614Q, I801T, N841S and Q885E). In addition, no frame shifts, deletions, insertions or recombination events were observed in the S gene sequences from any strain identified in the present study.

## Discussion

Similar to other countries and regions in the world, diarrhoea is one of the most common disease symptoms in calves in China, leading to great economic losses. The financial losses arise not only from mortality but also from the cost of medication (especially antimicrobials), the labour needed to treat sick calves, the reduced growth of calves, and the increased age and difficulty at first calving. Calf diarrhoea has a multifactorial aetiology in which viruses, bacteria, protozoa, and environmental factors (feeding, housing, and hygienic conditions) play a role. Recently, several viral pathogens were identified to be associated with calf diarrhoea, such as BToV, BNoV, BNebV, BAstV, BVDV, BKV, BRV and BCoV. Unusually noticeably, BToV and BCoV are two distinct viruses and they have different sequences. And the preliminary identification was based on the primers designed according to their respective conservative regions, without mutual interference. In addition, the detection rate of BCoV is much higher than that of BToV in all clinic cases (results not shown) in our study.

BToV is a viral pathogen of calves that causes diarrhoea and respiratory tract infections in calves and adult cattle and is globally responsible for serious economic losses in cattle production. However, BToV is usually not included in algorithms used to diagnose calf diarrhoea. To date, BToV has been described in diarrhoeic calves in various countries [4, 12–27]. According to a previous study [29], BToV alone has been shown to act as an enteric pathogen in cattle, and has also been regarded as a common relevant pathogen of cattle in the European and American cattle industries. Also, BToV can be identified in feces from clinically healthy caves, although there was a significant greater number positive samples in the diarrheic calves than in the healthy calves (*P* < 0.01) [20], which makes assessing the clinical relevance of BToV very difficult and the role of BToV in calf diarrhea still remains undetermined. However, we believe that our study will, to some extent, help to clarify the role of BToV in diarrhoea in calves. BRV, BCoV and BVDV are considered the major diarrhoea-inducing pathogens in China [30]. Notably, three out of eight BToV positive samples detected only BToV in our study, the other five samples were tested and positive for some other diarrhea pathogens. The results of pathogenic detection may indicated that BToV is a relevant pathogen in cattle, and it may also play a synergistic role in coinfections [23]. However, to date, little attention has been given to BToVs as causes of calf diarrhoea in China.

The percentage of BToV infection found in this study (1.74%) is comparatively lower than that reported in other studies: 2.9% in South Korea [22], 4.7% in Turkey [24], 5.2% in Austria [19], 6.25% in Brazil [23], 8.4% in Japan [21], 9.7% in the USA [18], 36.4% in Canada [4], and 43.2% in Croatia [26]. Despite these differences, one cannot make a reliable comparison among these percentages because of the limited sample size used in the present and previous studies. Interestingly, the eight positive samples were all obtained from Henan Province. No positive samples were obtained from Sichuan and Jiangsu Provinces, and no positive samples were detected among the yak samples. This may be due to geographic location, breed and feeding mode. These results showed that BToV is present in cattle in China. Our findings have significant implications for the diagnosis and control of calf diarrhoea in China and reveal the necessity for the development of prevention and control strategies, including the study of vaccine potency and the development of therapies. Previously, BToV-associated diarrhoea has not been reported in China, and no information about BToV epidemiology was available. Therefore, the epidemiology of BToV in China needs to be further evaluated.

The S protein is responsible for the antigenic properties of the virus and contains the binding site for the cell-surface receptor. Compared with the S genes of BToV strains identified from other countries, the Chinese BToV S genes shared a high nt/aa identity sequence homology with the S genes of Japanese BToV (94.3–97.1%/95.1–97.9%), Turkey BToV (95.9–97.7%/96.1–98.4%), Dutch BToV (95.5–97.3%/96.2–98.2%) and American BToV (Breda 1 strain, the first BToV detected in the USA in 1982) (95.2–96.2%/96.3–97.2%). The S genes of the newly identified Chinese strains are more closely related to those of the Turkish BToVs than those of the Japanese BToVs. Moreover, the Chinese BToVs shared low nucleotide identity with non-bovine ToV strains, such as human, equine, and porcine strains. Phylogenetic analysis based on the complete S gene sequences showed that the eight newly identified Chinese BToV strains were clustered into two different subclades, as described above. Comparison of the nt and deduced aa sequences of the complete S genes of the Chinese BToV strains showed that they were highly conserved in this region. Although the molecular characterization revealed some nt and aa differences between the strains identified in this study and the reference strains, the Chinese sequences were generally clustered with the reference strains. In this study, S protein was chosen for analysis because it may play an important role in pathogenesis. In fact, there are very few reports on these, therefore no more analysis has been done in this article. However, we believe that with the gradual deepening of research on BToV, these will become clearer.

Coronaviruses have been studied in detail, but little is known about ToVs. One important reason for this gap in knowledge is that ToVs have not been propagated in cell culture, with the sole exception of the Berne virus. However, research on this virus has been limited partly because of insufficient information and the lack of an available in vitro system for conducting further studies. Limited genomic information has also restricted our understanding of the virus. This has hampered the detailed characterization of the molecular biology, evolution, and taxonomy of this virus. Therefore, additional sequence information is expected to facilitate further research on virus antigenicity, virulence, infection, and replication in animals. This study will improve the understanding of the epidemiology, evolutionary pattern and genetic diversity of BToVs.

## Conclusions

In conclusion, this study first demonstrated the presence of BToV in Chinese beef and dairy and suggested that two different subclades of BToV strains are circulating in China, which might pose a potential threat to the cattle industry in China. This study highlights the importance of continuous surveillance and the evaluation of the epidemiology and pathology of BToV in domestic cattle. These findings have implications for the diagnosis and control strategies used for this newly identified virus. Additionally, our findings will enhance the current understanding of the genetic evolution of BToV.

## Methods

### Faecal samples

A total of 461 faecal samples were collected from calves with diarrhoea from 38 farms in three intensive cattle farming regions of China from September 2017 to July 2019. These farms were in Henan (14 farms, *n* = 221), Jiangsu (three farms, *n* = 42) and Sichuan (21 farms, *n* = 198). These three provinces are the main cattle production areas in central, eastern and western China, and the geographical distance between the two most distant farms was > 2000 km. At each cattle farm, the samples were randomly collected from calves with signs of clinical diarrhoea, and were directly collected with sampling bags during defecation by ourselves or farm veterinarian, the sampling requirements are the same. The ages of the tested calves ranged from 2 days to 4 months. All samples were transported on ice and stored at − 80 °C.

### RNA extraction and cDNA synthesis

The faecal samples were fully resuspended in phosphate-buffered saline (1:10 w/v) and centrifuged at 10,000 g for 10 min at 4 °C, followed by filtration through a 0.22 μm filter. Total RNA was extracted from 200 μL of the faecal suspension using the TaKaRa MiniBEST Viral RNA/DNA Extraction Kit Ver. 5.0 (TaKaRa Bio Inc.) according to the manufacturer’s instructions, and the cells were maintained at − 80 °C. cDNA was synthesized using the PrimeScript II 1st Strand cDNA Synthesis Transcription Kit (TaKaRa Bio Inc.) according to the manufacturer’s instructions and stored at − 20 °C.

### Detection of BToVs

BToVs were detected by reverse transcription polymerase chain reaction (RT-PCR) targeting a 603 bp fragment of the M gene, as previously reported [31]. PCR amplification was conducted in a 20 μL reaction volume containing 1.0 μL forward primer (10.0 μM), 1.0 μL reverse primer (10.0 μM), 2.0 μL cDNA, 10.0 μL Premix Taq (TaKaRa Taq Version 2.0) (TaKaRa Bio Inc.) and an appropriate volume of double-distilled water. The PCR conditions were as follows: 35 cycles of 94 °C for 30 s, 49 °C for 40 s and 72 °C for 50 s, followed by a final extension at 72 °C for 5 min. For each reaction, sterile ddH_2_O was used as a negative control. All of the RT-PCR products were verified by sequencing at Sangon Biotech (Zhengzhou, China).

To investigate coinfection with other pathogens, the eight BToV-positive samples were subjected to previously described specific RT-PCR assays for BNoV, BCoV, BRV (A, B, C), BVDV, BKV, BAstV and BNebV (The information of primers see Table S1). The faecal samples were also tested for *Coccidium* species and *Cryptosporidium* species using the sucrose floatation method. At the time of sampling, all the 8 BToV-positive cattle had been treated with antibiotics (including sulfonamides, penicillin, cephalosporin, and enrofloxacin, which were less efficient), so no bacterial isolation and identification was carried out.

### Amplification and sequencing of the S gene

Four pairs of overlapping primers were designed (reference sequence accession number: MG957146) to amplify the complete S gene according to the BToV sequences available in GenBank (Table 3). All amplification products were purified using the Omega Gel kit (Omega) by following the manufacturer’s instructions, after which the products were ligated into the pMD18-T simple vector (TaKaRa Bio, Inc.) and transformed into DH5α competent *Escherichia coli* cells (TaKaRa Bio Inc.). For each product, three to five colonies were selected and sequenced (Sangon, China) in both directions. The sequences were assembled using SeqMan software (version 7.0; DNASTAR Inc., WI, USA).

**Table 3:** Primer pairs used to amplify the partial M and full-length S genes

Primer Name	Sequence (5^′^-3^′^)	Position	Size (bp)
MF	TTCTTACTACACTTTTTGGA	M gene 98–117	603
MR	ACTCAAACTTAACACTAGAC	M gene 681–700	
S1F	ATGTTTTTATGTCTCTGTACCGCG	S gene1–24	1481
S1R	GCTGCAGCAATAGACCACATAGAC	S gene 1458–1481	
S2F	AGAATTGAACAACAGACTGGTGAC	S gene 1351–1374	1247
S2R	GGCACTTCATCAATTGGTACAT	S gene 2576–2597	
S3F	CCATCTGGTTGTCCTGTCCG	S gene 2464–2483	1433
S3R	ATGCACCATAGCATCAGTCAC	S gene 3877–3897	
S4F	AACCAATTTTTTCAGAGCGTGAG	S gene 3827–3849	929
S4R	CTAGCTTTTCTTAACCTTGC	S gene 4736–4755	

### Homology, phylogenetic, and recombination analyses

Sixteen reference sequences of the complete S gene from different ToV strains were downloaded from GenBank for use in the downstream analyses. The homologies of the nt and deduced aa sequences were determined using the MegAlign module in DNASTAR 7.0 software (DNASTAR Inc.). Multiple sequence alignment was performed, and subsequently, a neighbour-joining tree was constructed with 1000 bootstrap replicates in MEGA 7.0. Recombination events were assessed using SimPlot software (version 3.5.1) and RDP 4 software with the RDP, GeneConv, Chimaera, MaxChi, BootScan, SiScan, and 3Seq methods [32].

## Supplementary information

**Additional file 1 : Table S1.** Primers used for the detection of viruses in fecal samples from diarrheic calves.

## Data Availability

The datasets generated and/or analysed during the current study are available in the GenBank repository (MT364036 to MT364043). The data are simultaneously made available to ENA in Europe and the DNA Data Bank of Japan. All data generated or analyzed during this study are included in this published article and its additional files, and the dataset analyzed during the current study is available from the corresponding author on reasonable request.

## Abbreviations

BAstV: bovine astrovirus
BCoV: bovine coronavirus
BKoV: bovine kobuvirus
BNoV: bovine norovirus
BRoV: bovine rotavirus
BToV: bovine torovirus
BVDV: bovine viral diarrhea virus
ToV: torovirus
