# Supplementary material for: First report and genetic characterization of bovine torovirus in diarrhoeic calves in China
Source: BMC Vet Res. 2020 Aug 5;16:272. doi: 10.1186/s12917-020-02494-1 (PMC7404080; doi:10.1186/s12917-020-02494-1)
Supplement: Supplementary file 1 — Additional file 1 : Table S1. Primers used for the detection of viruses in fecal samples from diarrheic calves. [file 12917_2020_2494_MOESM1_ESM.docx]

**Table S1** Primers used for the detection of viruses in fecal samples from diarrheic calves

| Virus | Sequence (5^’^-3^’^) | Amplicon size (bp) |
| --- | --- | --- |
| BNoV | F: AGTTAYTTTTCCTTYTAYGGBGA | 532 |
|  | R: AGTGTCTCTGTCAGTCATCTTCAT |  |
| BCoV | F: GCAATCCAGTAGTAGAGCGT | 700 |
|  | R: CTTAGTGGCATCCTTGCCAA |  |
| BRV A | F: GCCTTTAAAAGCGAGAATTT | 1060 |
|  | R: GGTCACATCATACAAYTC TA |  |
| BRV B | F: GGAAATAATCAGAGATG | 795 |
|  | R: CTACTCGTTTGGCTCCCTCC |  |
| BRV C | F: TCAAGAAATGGWATGCAACC | 585 |
|  | R: CATAGCMGCTGGTCTWATCA |  |
| BVDV | F: GCTAGCCATGCCCTTAG | 290 |
|  | R: CCATGTGCCATGTACAG |  |
| BKoV | F: TGGAYTACAAGRATGTTTTGATGC | 216 |
|  | R: TGTTGTTRATGATGGTGTTGA |  |
| BAstV | F: GAYTGGACBCGHTWTGATGG | 432 |
|  | R: KYTTRACCCACATNCCAA |  |
| BNebV | F: CAGCCCGTCTGGGTGAAT | 524 |
|  | R: CCAGCGTTAGCGTTCCAG |  |

^a^ F: forward primer for RT-PCR; R: reverse primer for RT-PCR.
